# Claudin-10 is a new candidate prognostic marker in metastatic high-grade serous carcinoma

**DOI:** 10.1007/s00428-023-03541-6

**Published:** 2023-04-17

**Authors:** Ben Davidson, Delfim Doutel, Arild Holth, Dag Andre Nymoen

**Affiliations:** 1 Department of Pathology, Oslo University Hospital, Norwegian Radium Hospital, Montebello N-0310, Oslo, Norway; 2 Faculty of Medicine, Institute of Clinical Medicine, University of Oslo, N-0316 Oslo, Norway; 3 Present Address: Instituto Português de Oncologia de Lisboa Francisco Gentil, Serviço de Anatomia Patológica, R. Prof. Lima Basto 1099-023, Lisbon, Portugal; 4 Present Address: Department of Medical Genetics, Oslo University Hospital, Norwegian Radium Hospital, N-0310 Oslo, Norway

**Keywords:** Claudin-10, Quantitative RT-PCR, Immunohistochemistry, High-grade serous carcinoma, Mesothelioma, Effusion

## Abstract

The objective of this study was to analyze the expression and prognostic role of the tight junction protein claudin-10 in high-grade serous carcinoma (HGSC). Claudin-10 protein expression by immunohistochemistry was analyzed in 588 HGSC (414 effusions, 174 surgical specimens). Expression in mesotheliomas (n = 97; 47 effusions, 50 surgical specimens) was studied for comparative purposes. *CLDN10* mRNA expression by quantitative RT-PCR (qRT-PCR) was analyzed in 40 HGSC effusions. Claudin-10 protein expression was found in 360/588 (61%) HGSC vs. 19/97 (20%) mesotheliomas (p < 0.001), and was higher in HGSC surgical specimens compared to effusions (p < 0.001). qRT-PCR confirmed the presence of *CLDN10* mRNA in HGSC effusions. High (> 25%) claudin-10 expression in HGSC effusions was significantly associated with shorter overall survival (OS; p = 0.036) and progression-free survival (PFS; p = 0.045) in univariate analysis, and was an independent prognosticator of OS in multivariate analysis (p = 0.045). In conclusion, claudin-10 protein expression is higher in HGSC compared to mesothelioma, although the diagnostic power of this marker appear to be lesser than other claudin family members. Claudin-10 expression in HGSC effusions is marker of more aggressive disease.

**Supplementary Information:**

The online version contains supplementary material available at 10.1007/s00428-023-03541-6.

## Introduction

Ovarian cancer accounted for 313,959 new diagnoses and 207,252 deaths in 2020, making it the 8^th^ most common and the 8^th^ most lethal cancer in women [1]. The majority (> 90%) of ovarian cancers are tubo-ovarian carcinomas, of which high-grade serous carcinoma (HGSC) is the most common, accounting for 70% of cases [2].

Mesothelioma, a primary neoplasm of the serosal cavities, is a rarer cancer, which accounted for 30,870 cancer diagnoses and 26,278 deaths in both genders in 2020, with the corresponding figures for females at 9,310 and 7,597, respectively [1]. While more common in the pleura, the disease also affects the peritoneal cavity, where the percentage of women diagnosed with the disease among all patients is higher than in the pleural cavity.

In the absence of effective screening for either of these cancers, many patients are diagnosed with advanced-stage disease. The clinical presentation of HGSC and mesothelioma in the abdominal cavity, characterized by peritoneal dissemination, often in the presence of malignant ascites, may be indistinguishable. These tumors additionally have common expression of some markers, including cytokeratins, vimentin, epithelial membrane antigen (EMA), CA 125, WT1, D2-40 and mesothelin [3].

Claudins are a family of tight junction-specific membrane proteins that are present in epithelial and endothelial cells. Their expression pattern varies across different cell and tissue types, with at least two claudin members found in cells of most tissues. Claudins are often deregulated in cancer, and claudins 3 and 4, receptors of the *Clostridium perfringens* enterotoxin, among the most frequently members whose expression is altered in cancer [4, 5].

Several claudin family members have been shown to be overexpressed in HGSC compared to mesothelioma in gene expression array analysis [6], and several studies have reported on the overexpression of for claudins 1, 3, 4, 5 and 7 proteins in adenocarcinomas of various origin compared to mesothelioma and/or reactive mesothelial cells (RMC) in effusions [7–12]. Expression of claudins 1, 3 and 7 in tubo-ovarian carcinoma effusions was reported to be associated with shorter survival [13].

*CLDN10* gene expression has been reported to be associated with poor survival in three studies of public databases [14–16]. One of the latter studies additionally analyzed an in-house series which has not been reviewed based on the 2014 WHO guidelines and included, in addition to carcinomas, tumors labeled ‘germ cell’ and ‘other’ [16]. Data regarding the diagnostic and prognostic role of claudin-10 in a series of well-characterized in-house tumors is consequently lacking to date. The objective of the present study was to investigate these issues.

## Material and methods

### Patients and specimens

#### HGSC

HGSC specimens consisted of 588 tumors (414 effusions, 174 surgical specimens). The 414 effusions were from 414 patients and consisted of 356 peritoneal and 56 pleural specimens submitted to the Department of Pathology at the Norwegian Radium Hospital during the period of 1998 to 2017. Among the 406 specimens with known treatment data, 273 were primary diagnosis (chemo-naïve) effusions and 133 were post-neoadjuvant effusions, the majority tapped at disease recurrence.

The 174 surgical specimens were from 109 patients (51 patients with 1 lesion, 51 with 2 lesions and 7 with tumors from 3 anatomic locations) and consisted of ovarian, peritoneal and omental tumors resected in the period 1996–2007. The majority of the latter were not patient-matched with respect to effusions. Among the 71 patients with full clinical data, from whom a total of 104 surgical specimens were analyzed, 50 received upfront surgery and 21 were operated post-neoadjuvant chemotherapy. As the SEE-FIM procedure has not been applied during this period, specimens are referred to as HGSC, without reference to primary vs. metastatic location, though the latter is assumed for the majority.

A series of pleural and peritoneal mesotheliomas (n = 97; 47 effusions, 50 surgical specimens) from patients diagnosed with epithelioid or biphasic mesothelioma, confirmed in biopsy specimens, was studied for comparative purposes. Specimens were submitted to the Department of Pathology at the Norwegian Radium Hospital during the period of 1986 to 2008.

Effusions were centrifuged immediately after tapping, and cell pellets were frozen at -70 °C in equal amounts of RPMI 1640 medium (GIBCO-Invitrogen, Carlsbad, CA) containing 50% fetal calf serum (PAA Laboratories GmbH, Pasching, Austria) and 20% dimethylsulfoxide (Merck KGaA, Darmstadt, Germany). Cell blocks were prepared using the thrombin clot protocol. Tumors were diagnosed by an experienced gynecologic pathologist and cytopathologist (BD) based on established guidelines [2, 3].

Clinicopathologic data for the HGSC effusion cohort are detailed in Table 1.

**Table 1:** Clinicopathologic parameters of the HGSC effusion cohort (n = 414)

Parameter	Distribution
Age (mean)	23–88 years (62)
FIGO stage	
I	4
II	7
III	240
IV	155
NA	8
Residual disease	
0	67
≤ 1 cm	130
> 1 cm	128
NA	89
CA 125 at diagnosis (range; median)^*a*^	10–62,400 (1200)
Chemoresponse after primary treatment	
CR	192
PR	102
SD	32
PD	38
NA^*b*^	50

*NA* not available, *CR* complete response, *PR* partial response, *SD* stable disease, *PD* progressive disease

^*a*^ Available for 325 patients

^*b*^ Not available (missing data or disease response after chemotherapy could not be evaluated because of normalized CA 125 after primary surgery or missing CA 125 information and no residual tumor)

### IHC

Formalin-fixed, paraffin-embedded sections from the above-described 685 tumors were analyzed for claudin-10 protein expression using the Dako EnVision Flex + System (K8012; Dako, Glostrup, Denmark). The claudin-10 antibody was a rabbit polyclonal antibody purchased from Invitrogen (cat # 38–8400; Waltham, MA), applied at a 1:100 dilution following antigen retrieval in LpH buffer (pH 6.0).

Following deparaffinization, sections were treated with EnVision™ Flex + mouse linker (15 min) and EnVision™ Flex/HRP enzyme (30 min) and stained for 10 min with 3′3-diaminobenzidine tetrahydrochloride (DAB), counterstained with hematoxylin, dehydrated and mounted in Toluene-Free Mounting Medium (Dako). Positive control consisted of normal pancreas. In negative controls, the primary antibody was replaced with rabbit serum diluted to the same concentration as the primary antibody.

#### IHC scoring

Staining was scored by a surgical cytopathologist (DD), using a 0–4 scale as follows: 0 = no staining, 1 = 1–5%, 2 = 6–25%, 3 = 26–75%, 4 = 76–100% of tumor cells.

### qRT-PCR

*CLDN10* mRNA expression was analyzed in 40 HGSC effusions from the series analyzed using IHC. Effusions were centrifuged to obtain a cell pellet from which RNA was extracted using QIAsymphony (Qiagen, Hilden Germany). Details regarding reverse transcription, primer and probe design procedure and software, and efficiency testing were previously described [17]. The qRT-PCR reaction was run using the Perfecta qPCR ToughMix (Quanta Biosciences, Gaithersburg MD) and quantified on the Roche LightCycler 480 (Roche, Basel, Switzerland). Samples were analyzed in triplicate and average copy number was used for statistical analysis. *CLDN10* primer and probe sequences were as follows:

- Forward: GGGATTGTATTCATACTGTCAGGGC
- Reverse: CAAAGAGAGGATCAAAGAATTCCGT
- Probe: TGCTCAATGACTGGATGTTCCCTATATGCAAACAAAATC

The beta-glucuronidase (*GUS*), TATA box binding protein (*TBP*) and mitochondrial ribosomal protein L19 (*MRLP19*) genes were used as reference genes following previous testing [17] applying established guidelines [18–20]. Primer and probe sequences were previously detailed [17].

### Statistical analysis

Statistical analysis was performed applying the SPSS-PC package (Version 28). Probability of < 0.05 was considered statistically significant.

The Mann–Whitney U test was applied to comparative analyses of claudin-10 protein expression by IHC in HGSC vs. mesothelioma, as well as between HGSC effusions and surgical specimens.

The Mann–Whitney U test or the Kruskal–Wallis H test was applied to analysis of the association between claudin-10 protein expression by IHC and clinicopathologic parameters (for 2-tier or 3-tier analyses, respectively) for patients with HGSC effusions. For this analysis, clinicopathologic parameters were grouped as follows: age: ≤ 60 vs. > 60 years; effusion site: peritoneal vs. pleural; FIGO stage: III vs. IV; chemotherapy status: pre- vs. post-chemotherapy specimens; residual disease (RD) volume: 0 cm vs. ≤ 1 cm vs. > 1 cm; response to chemotherapy: complete response vs. partial response/stable disease/progressive disease.

Progression-free survival (PFS) and overall survival (OS) were calculated from the date of the last chemotherapy treatment/diagnosis to the date of recurrence/death or last follow-up, respectively. Univariate survival analyses of PFS and OS were executed using the Kaplan–Meier method and log-rank test. Platinum resistance was defined as PFS ≤ 6 months according to guidelines published by the Gynecologic Oncology Group (GOG) and progressive disease or recurrence was evaluated by the *Response Evaluation Criteria In Solid Tumors* (RECIST) criteria. In this analysis, claudin-10 protein expression was grouped as high vs. low based on cut-off at 25%, while the median value was used for *CLDN10* mRNA.

## Results

**Fig. 1:**
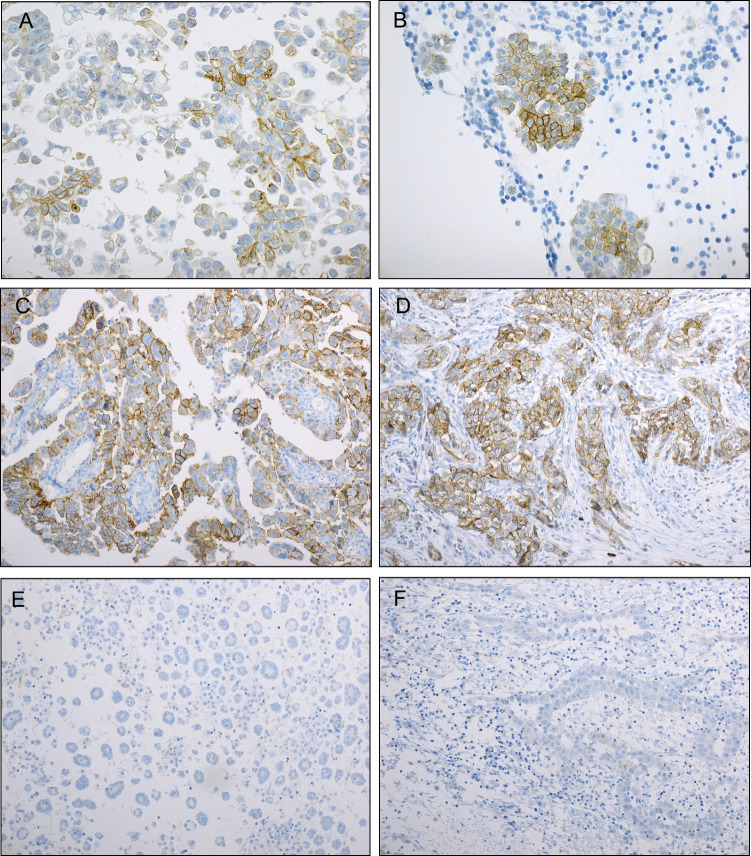
Immunohistochemistry. (**A**-**D**) Claudin-10 (CLD10) protein expression in high-grade serous carcinoma (HGSC). (**A**-**B**) Effusion specimens; (**C**-**D**) Surgical specimens. (**E**–**F**) CLD10 staining in mesothelioma. A CLD10-negative effusion is shown in Fig. 1-E, and a surgical specimen with focal (< 5%) fragmented staining is seen in Fig. 1-F

Claudin-10 protein expression was found in 360/588 (61%) HGSC vs. 19/97 (20%) mesotheliomas (Table 2, Fig. 1). High expression (> 25%) was observed in 80/414 (19%) HGSC effusions, 43/174 (25%) HGSC surgical specimens, 2/47 (4%) mesothelioma effusions and 1/50 (2%) mesothelioma biopsies/resections. Statistical analysis showed significantly higher claudin-10 expression in HGSC compared to mesothelioma (p < 0.001). Expression was additionally significantly higher in HGSC surgical specimens compared to effusions, with staining of any extent detected in 146/174 (84%) of the former vs. 214/414 (52%) of the latter (p < 0.001). This difference was not observed between mesothelioma effusions and solid specimens (p = 0.424). qRT-PCR confirmed the presence of *CLDN10* mRNA in all HGSC effusions.

**Table 2:** Claudin-10 expression in HGSC and mesothelioma specimens

Effusion status	Staining extent (% of cells)					
	0%	1–5%	6–25%	26–75%	76–100%	p-value
HGSC effusions (n = 414)	200	82	52	49	31	p < 0.001^*a*^
HGSC surgical specimens (n = 174)	28	54	49	29	14	
Mesothelioma effusions (n = 47)	36	9	0	1	1	
Mesothelioma surgical specimens (n = 50)	42	4	3	1	0	

^*a*^ For the comparison between HGSC and mesothelioma and between HGSC effusions and surgical specimens

Claudin-10 protein expression in HGSC effusions was significantly higher in specimens from younger patients (p = 0.013) and showed a trend towards association with primary chemoresistance (p = 0.056). It was unrelated to effusion site (p = 0.75), previous chemotherapy (p = 0.728), FIGO stage (p = 0.866), RD volume (p = 0.587) or chemotherapy response at diagnosis (p = 0.509).

*CLDN10* mRNA expression in HGSC effusions was unrelated to age (p = 0.738), effusion site (p = 0.28), previous chemotherapy (p = 0.537), FIGO stage (p = 0.688), RD volume (p = 0.827) or primary chemoresistance (p = 0.469). However, it was significantly higher in specimens from patients who had unfavorable (non-complete) response to chemotherapy at diagnosis (p = 0.035).

Clinical data were available for 71 of the 109 patients with surgical HGSC specimens. No association was observed between claudin-10 protein expression and patient age (p = 0.863), FIGO stage (p = 0.181), RD volume (p = 0.693) or chemotherapy response at diagnosis (p = 0.989). However, expression was higher in specimens from patients who received neoadjuvant chemotherapy (21/21 expressing claudin-10, 8 with expression > 25%) compared to those who received upfront surgery (38/50 expressing claudin-10, 12 with expression > 25%; p = 0.033).

**Fig. 2:**
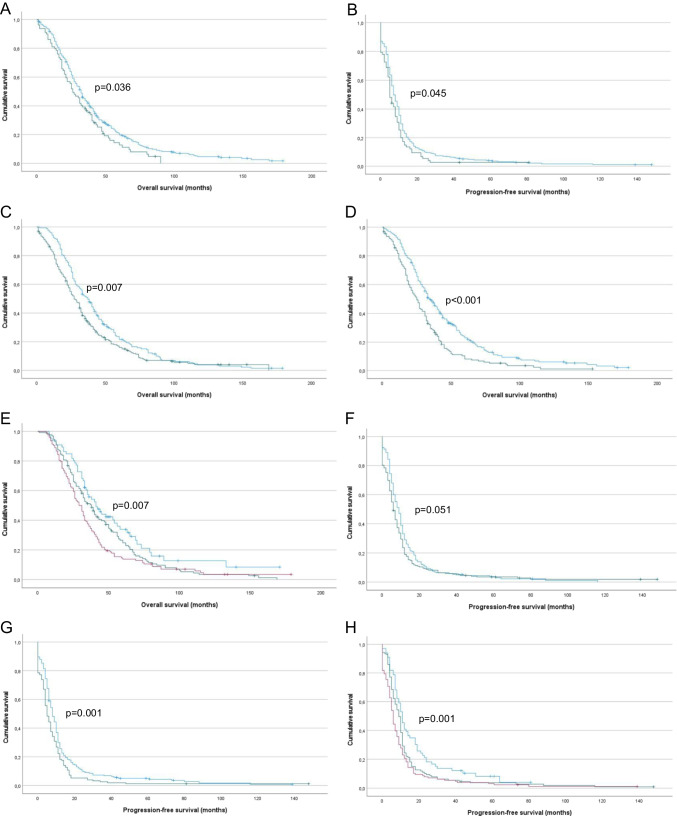
Survival. **A:** Kaplan–Meier survival curve showing the association between CLD10 protein expression and overall survival (OS) for 409 HGSC patients with malignant effusion. Patients with effusions with high (> 25%) CLD10 expression (n = 79; green line) had mean OS of 32 months compared to 42 months for patients with effusions showing low (≤ 25%) expression (n = 330, blue line; p = 0.036). **B:** Kaplan–Meier survival curve showing the association between patient age and OS for 409 HGSC patients. Older (> 60 years) patients (n = 234; green line) had mean OS of 37 months compared to 45 months for younger (≤ 60 years) patients (n = 175, blue line; p = 0.007). **C:** Kaplan–Meier survival curve showing the association between FIGO stage and OS for 394 HGSC patients with advanced-stage disease. Patients diagnosed with stage IV disease (n = 155; green line) had mean OS of 31 months compared to 45 months for patients with stage III disease (n = 239, blue line; p < 0.001). **D:** Kaplan–Meier survival curve showing the association between residual disease (RD) volume and OS for 324 patients with debulking data. Patients debulked to no macroscopic disease (n = 66; blue line) had mean OS of 57 months compared to 46 and 39 months for patients debulked to 1 cm (n = 130, green line) and ≥ 2 cm (n = 128, red line), respectively (p = 0.007). **E:** Kaplan–Meier survival curve showing the association between CLD10 protein expression and progression-free survival (PFS) for 394 HGSC patients with PFS data. Patients with effusions with high (> 25%) CLD10 expression (n = 77; green line) had mean PFS of 9 months compared to 13 months for patients with effusions showing low (≤ 25%) expression (n = 317, blue line; p = 0.045). **F:** Kaplan–Meier survival curve showing the association between patient age and PFS for 394 HGSC patients. Older (> 60 years) patients (n = 222; green line) had mean PFS of 11.8 months compared to 13 months for younger (≤ 60 years) patients (n = 172, blue line; p = 0.051). **G:** Kaplan–Meier survival curve showing the association between FIGO stage and PFS for 381 HGSC patients with advanced-stage disease. Patients diagnosed with stage IV disease (n = 149; green line) had mean PFS of 9 months compared to 14 months for patients with stage III disease (n = 232, blue line; p = 0.001). **H:** Kaplan–Meier survival curve showing the association between residual disease (RD) volume and PFS for 319 patients with debulking data. Patients debulked to no macroscopic disease (n = 66; blue line) had mean PFS of 18 months compared to 14 and 11 months for patients debulked to 1 cm (n = 127, green line) and ≥ 2 cm (n = 126, red line), respectively (p = 0.001)

Survival data were available for 409 of the 414 patients with HGSC effusions in the IHC cohort, and the follow-up period ranged from 1 to 179 months (mean = 37 months, median = 31 months). PFS ranged from 0 to 148 months (mean = 11 months, median = 7 months). At the last follow-up, 370 patients were dead of disease, 22 were alive with disease and 9 were with no evidence of disease. Four patients died of complications and 1 patient died of unrelated causes. Three patients with initial follow-up were subsequently lost to follow-up. Higher (> 25%) claudin-10 protein expression was associated with significantly shorter OS (p = 0.036; Fig. 2-A) and PFS (p = 0.045; Fig. 2-B) in univariate survival analysis.

Among clinical parameters, older age (p = 0.007; Fig. 2-C), FIGO IV stage (p < 0.001; Fig. 2-D) and higher RD volume (p = 0.007; Fig. 2-E) were significantly related to shorter OS. In Cox multivariate survival analysis, in which these 4 parameters were entered, claudin-10 expression, FIGO stage and RD volume emerged as independent prognosticators (Claudin-10: p = 0.045; Age: p = 0.513; FIGO stage: p < 0.001; RD volume: p = 0.011).

When assessed for association with PFS, older age was only marginally related to shorter PFS (p = 0.051; Fig. 2-F), whereas FIGO IV stage (p = 0.001; Fig. 2-G) and higher RD volume (p = 0.001; Fig. 2-H) were significantly related to shorter PFS. In Cox multivariate survival analysis, only RD volume was an independent prognosticator (Claudin-10: p = 0.118; Age: p = 0.528; FIGO stage: p = 0.06; RD volume: p = 0.001).

In survival analysis that included the 40 patients in qRT-PCR cohort, higher *CLDN10* mRNA levels were associated with shorter OS, but not significantly (mean OS of 29 vs. 38 months; p = 0.214; data not shown). The same was true for the 71 patients with survival data in the cohort with surgical specimens (mean OS of 43 vs. 53 months; p = 0.282; data not shown).

In view of our recent and current observations regarding the prognostic role of claudins in this disease, we attempted to compare the prognostic role of claudin-10 in HGSC effusions to that of the previously-studied claudins 1, 3, 4 and 7 [13]. Only 104 specimens were analyzed both in the current and the previous study. In this smaller cohort, none of the claudins was significantly associated with OS or PFS. However, analysis limited to patients with post-chemotherapy effusions did show a trend for shorter overall survival for patients with tumors that had high claudin-1 and claudin-10 expression (p = 0.058 and p = 0.061, respectively; Supplementary Fig. 1).

## Discussion

The majority of studies that have focused on the diagnostic and clinical role of claudins in tubo-ovarian carcinoma predate the 2014 WHO classification and have therefore included tumors of different histotype. While it is likely that HGSC constituted the majority of cases in these series, type-specific studies provide more accurate information in this context.

The present study identified frequent expression of claudin-10 in HGSC, in agreement with previous studies of other claudin family members [7–13, 21–24]. However, in contrast to the previously reported overexpression of claudins 1, 3 and 7 in tubo-ovarian carcinoma effusions compared to surgical specimens [13], expression of claudin-10 was significantly higher in the latter. This may suggest changes in the claudin profile of HGSC cells in the anchorage-free, anoikis-resistant conditions of serous effusions. Beyond this, this suggests that claudin-10 is less robust than claudins 1, 3, 4 and 7 as a diagnostic marker of HGSC in the differential diagnosis from mesothelioma in serous effusions. In surgical specimens, claudin-10 performs better, as evidenced by expression in > 80% of tumors compared to 16% of mesotheliomas. Staining was nevertheless focal in the majority of cases, whereas expression of claudin-4, used in many laboratories in this diagnostic setting, is often more diffuse. Our data consequently do not support replacement of claudin-4 by claudin-10 for differentiating HGSC from mesothelioma, nor its precedence over claudins 1, 3 or 7 as a second marker.

Data regarding the clinical relevance of claudin-10 protein expression in HGSC is currently unavailable. However, several series that have focused on other claudins have been published. Expression of claudin-5 was associated with high grade, advanced stage and poor overall and disease-specific survival in analysis of ovarian specimens of serous carcinoma [21]. Claudin-7 protein expression was significantly related to poor chemotherapy response and shorter PFS in another study of ovarian specimens of various histotypes [22]. Claudin-4 expression was associated with significantly shorter PFS and OS in another study of ovarian specimens of various histotypes, though neither retained significance in multivariate analysis [23]. Conversely, claudin-4 expression was unrelated to PFS or OS in analysis of a larger series of HGSC [24].

Our group previously found significant association between expression of claudins 1, 3 and 7 in tubo-ovarian carcinoma effusions and shorter PFS and/or OS. Claudin-7 expression was an independent predictor of poor PFS in multivariate survival analysis of the entire cohort, whereas claudin-3 independently predicted poor OS in patients with postchemotherapy effusions [13].

In the present study, higher claudin-10 protein expression was significantly associated with shorter OS and PFS in univariate survival analysis, and this finding was retained in multivariate survival analysis of OS. Additionally, *CLDN10* mRNA expression in HGSC effusions was significantly related to unfavorable response to chemotherapy at diagnosis. This is in agreement with the majority of studies of other claudins in this disease, as well as data regarding *CLDN10* derived from public databases [14–16]. The higher expression of claudin-10 in surgical specimens obtained following neoadjuvant chemotherapy may suggest survival advantage for tumor cells expressing this protein.

In conclusion, claudin-10 is overexpressed in HGSC effusions and surgical specimens compared to mesothelioma, though with lesser sensitivity and specificity as diagnostic marker compared to other claudin family members, particularly in effusion specimens. The association between claudin-10 expression and poor outcome provides further support to previous observations linking this family to aggressive disease in tubo-ovarian carcinoma. One may speculate that the poor outcome associated with claudin expression is mediated through chemoresistance associated with the epithelial phenotype and/or may be linked to specific genomic signatures, including HRD, though validation of this hypothesis requires further research. Assessing the relative prognostic relevance of different claudins requires analysis of a large series analyzed simultaneously for several family members.

## Supplementary Information

Below is the link to the electronic supplementary material.

Supplementary Fig. 1 Claudins as prognosticators in post-chemotherapy effusions. A: Kaplan-Meier survival curve showing the association between CLD1 protein expression and OS for 47 HGSC patients with post-chemotherapy effusion. Patients with effusions with high (>25%) CLD1 expression (*n*=33; red line) had mean OS of 30 months compared to 43 months for patients with effusions showing low (≤25%) expression (*n*=14, blue line; *p*=0.058). B: Kaplan-Meier survival curve showing the association between CLD10 protein expression and OS for 47 HGSC patients with post-chemotherapy effusion. Patients with effusions with high (>25%) CLD10 expression (*n*=10; red line) had mean OS of 26 months compared to 36 months for patients with effusions showing low (≤25%) expression (*n*=37, blue line; *p*=0.061). (PPTX 64 KB)

## Data Availability

The datasets generated during and/or analysed during the current study are available from the corresponding author on reasonable request.
