# Correction to: Claudin-10 is a new candidate prognostic marker in metastatic high-grade serous carcinoma

**DOI:** 10.1007/s00428-023-03567-w

**Published:** 2023-06-01

**Authors:** Ben Davidson, Delfim Doutel, Arild Holth, Dag Andre Nymoen

**Affiliations:** 1 Department of Pathology, Oslo University Hospital, Norwegian Radium Hospital, Montebello, N-0310 Oslo, Norway; 2 Faculty of Medicine, Institute of Clinical Medicine, University of Oslo, N-0316 Oslo, Norway; 3 Instituto Português de Oncologia de Lisboa Francisco Gentil, Serviço de Anatomia Patológica, R. Prof. Lima Basto, 1099-023 Lisbon, Portugal; 4 Department of Medical Genetics, Oslo University Hospital, Norwegian Radium Hospital, N-0310 Oslo, Norway

**Correction to: Virchows Archiv**

10.1007/s00428-023-03541-6

The original published version of the above article contained an error. In the first sentence of the Abstract, originally written as ‘gap junction protein’, the correct terminology is ‘tight junction protein’.

The original article has been corrected.

