# Supplementary figures and images for: Claudin-10 is a new candidate prognostic marker in metastatic high-grade serous carcinoma
Source: Virchows Arch. 2023 Apr 17;482(6):975–82. doi: 10.1007/s00428-023-03541-6 (PMC10247576; doi:10.1007/s00428-023-03541-6)

## Slide 1
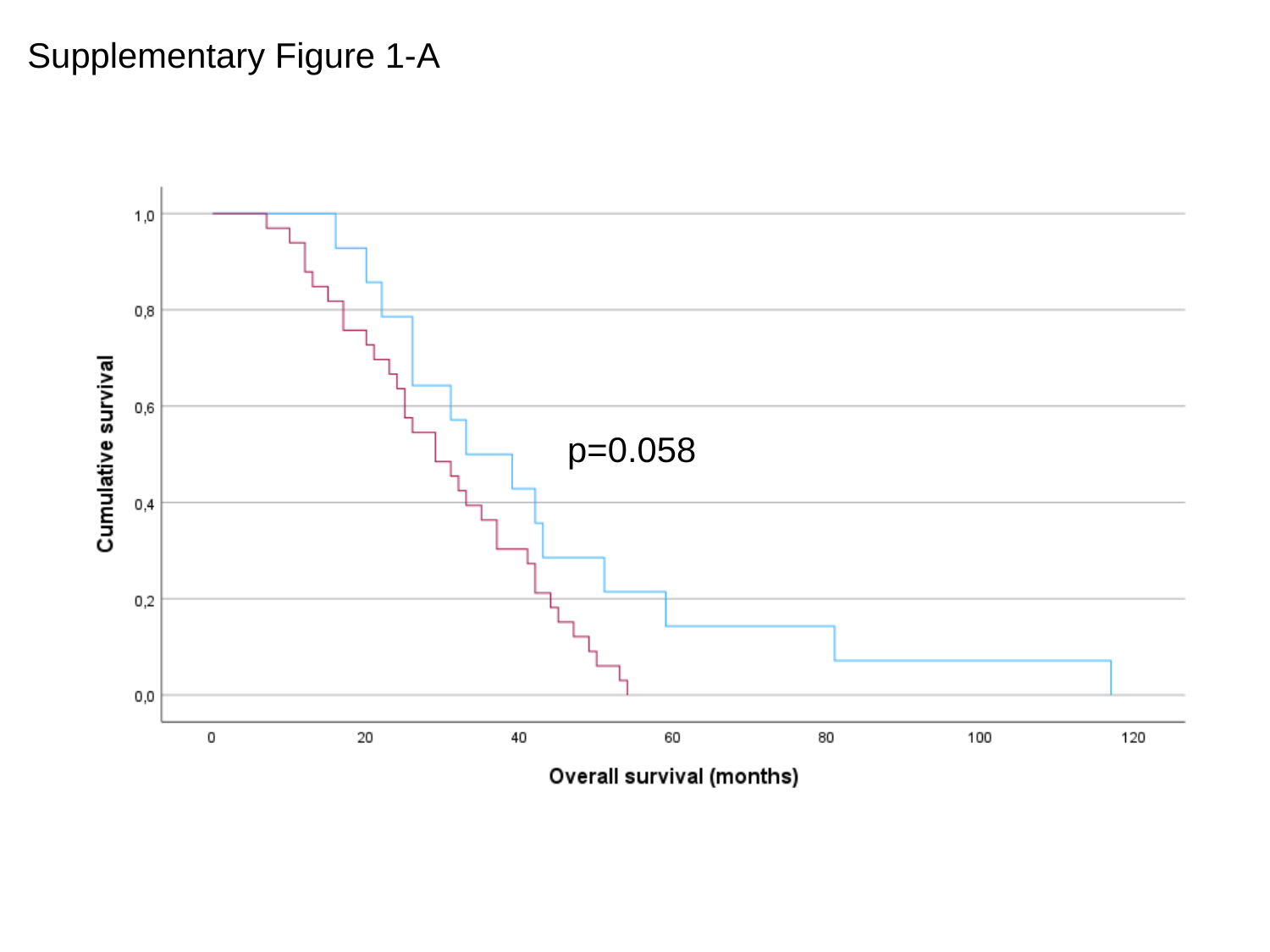

Supplementary Figure 1-A
p=0.058

## Slide 2
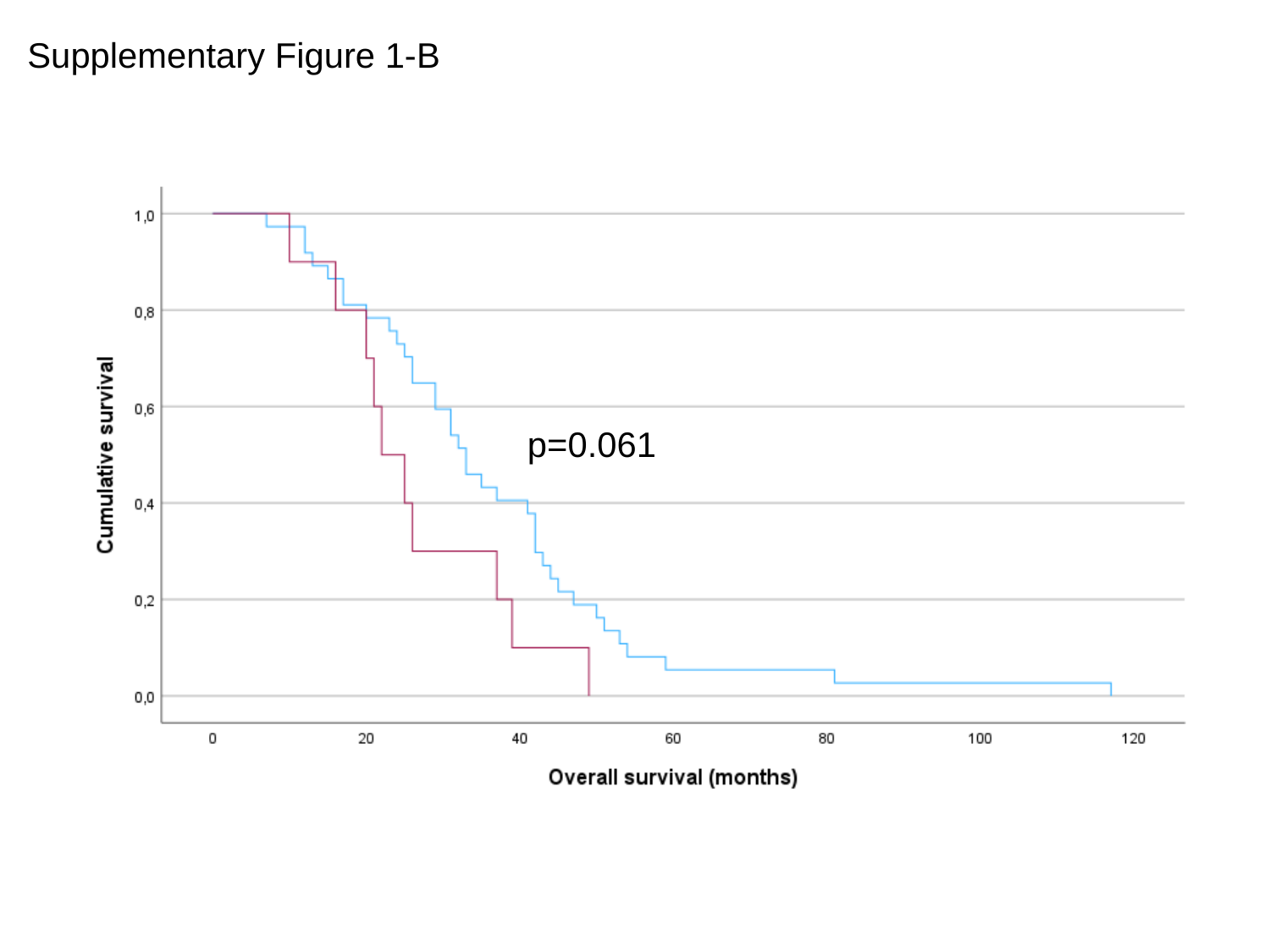

Supplementary Figure 1-B
p=0.061

Supplement: Supplementary file 1 — Claudins as prognosticators in post-chemotherapy effusions. A: Kaplan-Meier survival curve showing the association between CLD1 protein expression and OS for 47 HGSC patients with post-chemotherapy effusion. Patients with effusions with high (>25%) CLD1 expression (n=33; red line) had mean OS of 30 months compared to 43 months for patients with effusions showing low (≤25%) expression (n=14, blue line; p=0.058). B: Kaplan-Meier survival curve showing the association between CLD10 protein expression and OS for 47 HGSC patients with post-chemotherapy effusion. Patients with effusions with high (>25%) CLD10 expression (n=10; red line) had mean OS of 26 months compared to 36 months for patients with effusions showing low (≤25%) expression (n=37, blue line; p=0.061). (PPTX 64 KB) [file 428_2023_3541_MOESM1_ESM.pptx]
